# Enrichment of syngas‐converting communities from a multi‐orifice baffled bioreactor

**DOI:** 10.1111/1751-7915.12864

**Published:** 2017-11-21

**Authors:** Ana L. Arantes, Joana I. Alves, Alfons J. M. Stams, M. Madalena Alves, Diana Z. Sousa

**Affiliations:** ^1^ Centre of Biological Engineering University of Minho 4710‐057 Braga Portugal; ^2^ Laboratory of Microbiology Wageningen University Stippeneng 4 6708 WE Wageningen The Netherlands

## Abstract

The substitution of natural gas by renewable biomethane is an interesting option to reduce global carbon footprint. Syngas fermentation has potential in this context, as a diverse range of low‐biodegradable materials that can be used. In this study, anaerobic sludge acclimatized to syngas in a multi‐orifice baffled bioreactor (MOBB) was used to start enrichments with CO. The main goals were to identify the key players in CO conversion and evaluate potential interspecies metabolic interactions conferring robustness to the process. Anaerobic sludge incubated with 0.7 × 10^5^ Pa CO produced methane and acetate. When the antibiotics vancomycin and/or erythromycin were added, no methane was produced, indicating that direct methanogenesis from CO did not occur. *Acetobacterium* and *Sporomusa* were the predominant bacterial species in CO‐converting enrichments, together with methanogens from the genera *Methanobacterium* and *Methanospirillum*. Subsequently, a highly enriched culture mainly composed of a *Sporomusa* sp. was obtained that could convert up to 1.7 × 10^5^ Pa CO to hydrogen and acetate. These results attest the role of *Sporomusa* species in the enrichment as primary CO utilizers and show their importance for methane production as conveyers of hydrogen to methanogens present in the culture.

## Introduction

Biomass can be biologically converted to fuels and chemicals, but poorly biodegradable biomass (e.g. straw, wood) requires costly chemical or enzymatic hydrolysis prior to microbial fermentation. Gasification of biomass, and subsequent syngas fermentation, is an alternative that can maximize carbon recovery compared to conventional fermentations (Bredwell *et al*., 1999; Munasinghe and Khanal, 2010; Guiot *et al*., 2011). Syngas is mainly composed of CO, H_2_ and CO_2,_ and these compounds can be used by anaerobic microorganisms to produce value‐added chemicals. In the absence of external electron acceptors, CO can be converted by hydrogenogenic, acetogenic and methanogenic microorganisms (Diender *et al*., 2015). The hydrogenogenic conversion of CO results in the formation of H_2_ and CO_2_, and is typically performed by thermophilic carboxydotrophs, such as *Carboxydothermus hydrogenoformans*,* Moorella stamsii* and *Desulfotomaculum nigrificans* (Svetlitchnyi *et al*., 2001; Parshina *et al*., 2005; Henstra *et al*., 2007; Alves *et al*., 2013a; Visser *et al*., 2014; Diender *et al*., 2015). Carboxydotrophic mesophiles, such as *Clostridium carboxidivorans, C. ljungdahlii, C. autoethanogenum*,* Acetobacterium woodii*,* Sporomusa ovata* and *Butyribacterium methylotrophicum,* have the capability of converting CO into short‐chain fatty acids (mainly acetate) and alcohols (ethanol, butanol, 2,3‐butanediol) (Henstra *et al*., 2007; Balk *et al*., 2010; Köpke *et al*., 2010, 2011; Munasinghe and Khanal, 2011; Jang *et al*., 2012; Liu *et al*., 2014a,b; Diender *et al*., 2015). In general, CO is a poor substrate for methanogens. Methane production from CO has been reported only for some species of *Methanosarcina* and *Methanothermobacter* (Daniels *et al*., 1977; Rother and Metcalf, 2004; Henstra *et al*., 2007; Diender *et al*., 2016). For this reason, the utilization of mixed cultures (sludges) for the production of methane from syngas may be advantageous as normally these systems are more robust and less susceptible to inhibition (Guiot *et al*., 2011). Although methane production directly from CO is possible, hydrogenotrophic and acetoclastic methanogens can coexist with carboxydotrophic, hydrogenogenic and acetogenic bacteria and use the bacterial products (H_2_ and acetate) to ultimately produce methane. An additional advantage of using anaerobic sludge for syngas conversion is the possibility of implementing low‐cost, open fermentation systems. Methane is a direct substitute of natural gas, and infrastructure to distribute methane to industry and households is existing.

Bioreactor technology for open fermentation of syngas is also developing, with special attention to the requisites of high gas–liquid mass transfer rates and cell retention times. Several reactor types have been studied for syngas open fermentations, including gas‐lift reactors (Haddad *et al*., 2014), reverse membrane bioreactors (Youngsukkasem *et al*., 2015; Westman *et al*., 2016) and multi‐orifice baffled bioreactor (MOBB) (Pereira, 2014). The MOBB described by Pereira (2014) showed methane production rates of about 1.5–2 times higher than reported for the other systems. In this study, we analysed the microbial communities in the MOBB sludge and performed enrichment and microbial diversity studies to get more insight into key players and metabolic networks involved in CO biomethanation. The main aim was to verify whether methane production from CO occurs predominantly via direct methanogenesis or indirectly via bacterial–archaeal associations. Identifying key microbial interactions could explain and justify the higher robustness of mixed cultures for syngas conversion to methane.

## Results and discussion

Multi‐orifice baffled (bio)reactors (MOBB) are recognized for their excellent performance in gas–liquid mass transfer and therefore suited for the conversion of gaseous substrates (Ni *et al*., 2003). In a previous work, Pereira (2014) described the application of a MOBB to continuous syngas conversion to methane using open anaerobic mixed cultures (sludge). Although methane was efficiently produced from CO, the microorganisms involved in this conversion and possible metabolic interactions were not disclosed. In this study, we used a combination of enrichment studies and microbial diversity analyses to identify key microbial players and microbial interactions occurring in the MOBB sludge.

### CO to methane conversion by MOBB enrichments is dependent on bacterial–archaeal interactions

**Figure 1:**
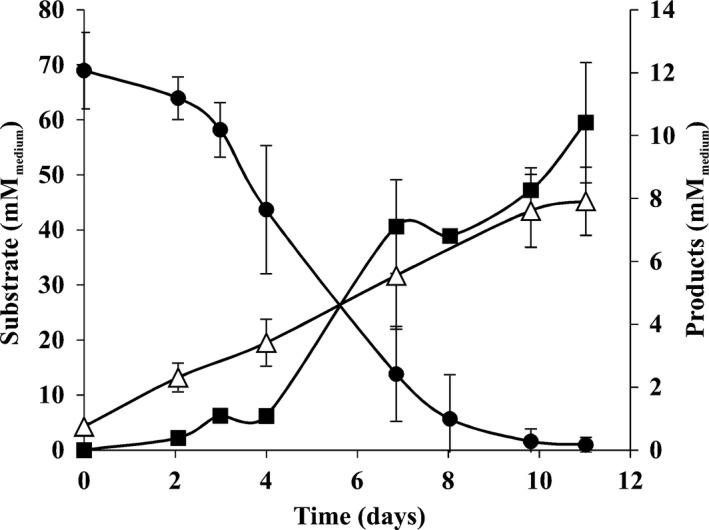
Substrate consumption and product formation by stable enrichment CO‐degrading cultures: CO(12) (after 12 successive transfers). Symbols: (●) carbon monoxide, (▵) acetate, (■) methane.

Incubation of MOBB sludge with 40% CO as sole carbon and energy source resulted in the production of methane and acetate. In Fig. 1, substrate consumption and product formation are shown for a stable enrichment culture obtained after 12 successive transfers of the MOBB sludge on CO (CO(12)): 68.9 mmol L^−1^_medium_ CO resulted in the formation of 10.4 mmol L^−1^_medium_ methane and 7.9 mmol L^−1^_medium_ acetate.

When the MOBB sludge was incubated in the presence of the antibiotics vancomycin and erythromycin (CO‐v_50_e_100_ and O‐CO‐v_100_e_100_), no CO conversion or methane production was observed over a long incubation period (over 3 months) (data not shown). Vancomycin and erythromycin are bacterial inhibitors and the fact that no CO conversion was observed in their presence indicates that CO was not directly metabolized by carboxydotrophic methanogens. Carbon monoxide is inhibitory for most methanogens, and only a few species can actually grow on this substrate: *Methanothermobacter thermoautotrophicus* (Daniels *et al*., 1977), *Methanothermobacter marburguensis* (Diender *et al*., 2016), *Methanosarcina barkeri* (O'Brien *et al*., 1984; Bott *et al*., 1986) and *Methanosarcina acetivorans* (Rother and Metcalf, 2004). Both thermophilic *Methanothermobacter* species grow significantly slower on CO than on H_2_/CO_2_ (Daniels *et al*., 1977; Diender *et al*., 2016). *Methanosarcina acetivorans* can withstand higher CO partial pressures than the hydrogenotrophic methanogens, but its methanogenic metabolism shifts towards formation of acetate and formate at increased CO pressures (Rother and Metcalf, 2004).

**Figure 2:**
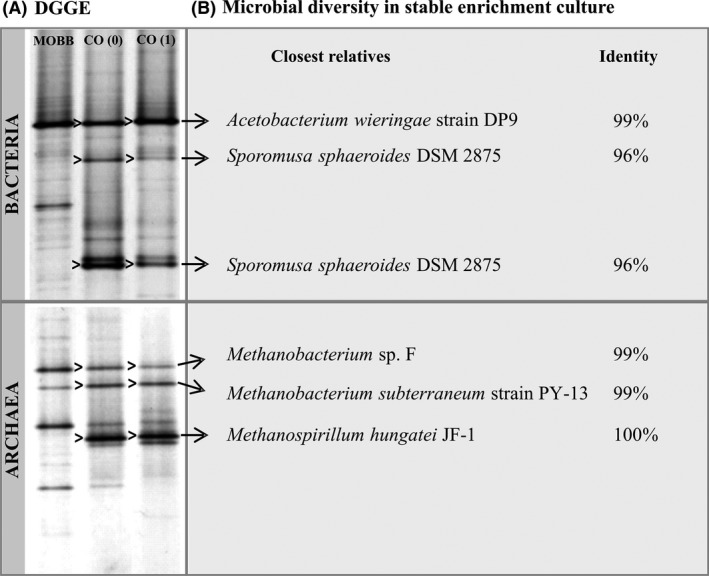
Microbial diversity in CO‐converting anaerobic enrichment (enrichment series CO(x)): (A) bacterial and archaeal DGGE profiles and (B) closely relative microorganisms of predominant clones obtained from the enrichment cultures. MOBB – inoculum sludge withdrawn from a MOBB fed with syngas; CO(0) and CO(1) – enrichment cultures incubated with CO as sole carbon and energy source, where (x) stands for number of successive transfers.

Analysis of the archaeal communities in the inoculum and initial CO enrichment cultures by cloning and sequencing (Sanger) revealed the presence of hydrogenotrophic methanogens closely related to *Methanobacterium* and *Methanospirillum* species (Fig. 2). However, microorganisms related to known carboxydotrophic methanogens were not detected, which again supports the hypothesis that methane was not directly produced from CO in these cultures. *Methanobacterium* and *Methanospirillum* species are capable of using H_2_/CO_2_ to produce methane (Whitman *et al*., 2006). Although H_2_ could not be detected in CO enrichments (Fig. 1), microbial H_2_ production from CO is possible (water–gas shift reaction, CO + H_2_O → H_2_ + CO_2_) (Diender *et al*., 2015). The two predominant bacteria identified in the enrichments were related to *Acetobacterium* and *Sporomusa* species (Fig. 2), which normally have a homoacetogenic metabolism when growing on CO (Genthner and Bryant, 1987; Balk *et al*., 2010; Diender *et al*., 2015). However, interspecies H_2_ transfer during growth of *Acetobacterium woodii* with different methanogens on sugars has been previously reported (Winter and Wolfe, 1980), indicating that a similar mechanism could take place during CO fermentation. Acetoclastic methanogens were not detected in the enrichment cultures (Fig. 2), and acetate accumulation was observed (Fig. 1). *Methanosarcina* and *Methanosaeta* species are commonly present in anaerobic sludges (Karakashev *et al*., 2005; Pan *et al*., 2016; Sancho Navarro *et al*., 2016), and the reason for their absence in the MOBB sludge is not clear. It might be that these microorganisms suffer from the shear stress caused by the oscillations in the MOBB. In addition, effects of CO toxicity towards aceticlastic methanogens cannot be excluded.

### A novel *Sporomusa* sp. is present in MOBB sludge that can convert CO to H_2_

From the DGGE profiles of cultures CO(0) and CO(1), it is evident that bacterial communities were dominated by *Acetobacterium* and *Sporomusa* species (Fig. 2). Carbon monoxide metabolism is well studied in *Acetobacterium woodii*. This organism was initially described to grow homoacetogenically on CO as a sole energy source (Genthner and Bryant, 1987), but it has been recently found that it can use CO only in cofermentation with H_2_ or formate (Bertsch and Müller, 2015). *Acetobacterium* population in the CO enrichments is most closely related to *Acetobacterium wieringae* (99% 16S rRNA gene identity). Recently, Sancho Navarro *et al*. (2016) reported the presence of bacterial species closely related to *A. wieringae* after incubation of anaerobic sludge at high CO concentrations. While only *A. woodii* is reported as carboxydotrophic organism, *A. wieringae* genome contains the gene clusters for both carbonyl and methyl branches of the Wood–Ljungdahl pathway, identical to what is found in *A. woodii* (Genthner and Bryant, 1987; Poehlein *et al*., 2016). *Acetobacterium* population became more predominant during the enrichment process (Fig. S1), and based on MiSeq results, it represented about 82% of the community in enrichment culture CO(12) (Table 1A).

**Table 1:** Microbial diversity of enrichments (A) CO(12) and (B) CO‐P(8)

	Closest relatives	Amount (%)	Coverage (%)^c^	Identity (%)^c^
(A)				
Bacteria	*Acetobacterium sp* (*Acetobacterium sp*. enrichment culture isolate DGGE gel band K1‐IRE21‐Sa 16S ribosomal RNA gene, partial sequence)^c^	82^a^	100	100
Archaea	*Methanospirillum sp* (*Methanospirillum hungatei* strain JF‐1 16S ribosomal RNA gene, complete sequence)^c^	7^a^	99	100
(B)				
Bacteria	*Sporomusa sp* (*Sporomusa ovata* strain DSM 2662 16S ribosomal RNA gene, partial sequence)^c^	97^b^	100	96
Archaea	*Caloramator quimbayensis* (*Caloramator quimbayensis* strain USBA A 16S ribosomal RNA gene, partial sequence)^c^	2^b^	98	97

a Percentage calculated based on a total number of counts of 28146.

b Percentage calculated based on a total number of counts of 41718.

c Results of sequence analysis on NCBI BLAST.

**Figure 3:**
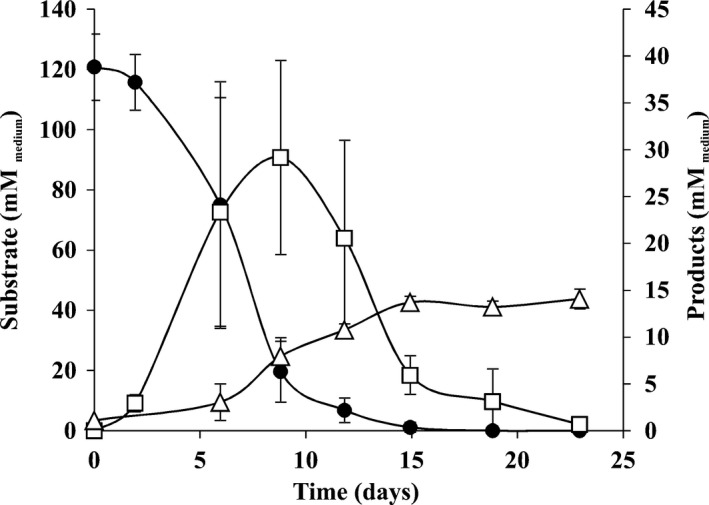
Substrate consumption and product formation by stable enrichment CO‐degrading cultures after pasteurization: CO‐P(8). Symbols: (●) carbon monoxide, (▵) acetate and (□) hydrogen.

**Figure 4:**
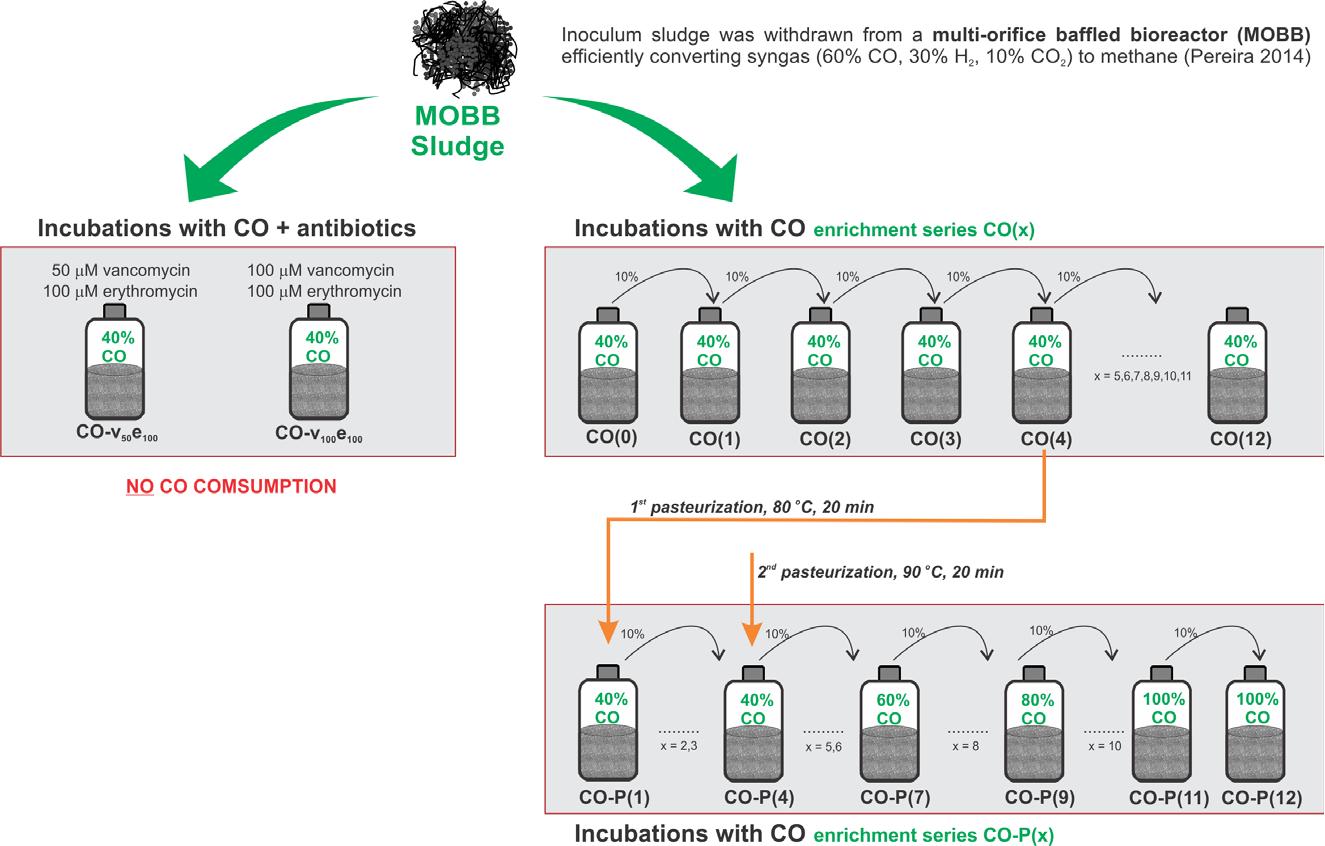
Experimental set‐up and identification of enrichment cultures promoted in this study. In the enrichment series CO(x) and CO‐P(x), (x) stands for the number of transfers over 2 years of period.

The presence of closely related organisms to *Sporomusa* species in the initial enrichments was rather striking. Carbon monoxide metabolism in *Sporomusa* species is poorly studied, although CO conversion to acetate has been reported for *S. termitida*,* S. ovata* and *Sporomusa* strain An4 (Breznak *et al*., 1988; Balk *et al*., 2010). *Sporomusa* strains are known to form spores (Möller *et al*., 1984), and it was clear from microscopic examination that spores were present in early CO enrichment cultures (data not shown). With the aim of enriching/isolating the *Sporomusa* species in the CO enrichments, we proceeded with the pasteurization of culture CO(4), and later on, a second pasteurization was performed with culture CO‐P(4) (see experimental set‐up in Fig. 4). Culture CO‐P(8) was highly enriched in *Sporomusa* (97% of the total microorganisms) (Table 1B) and was able to grow under a 100% CO headspace. Culture CO‐P(8)‐produced H_2_ from CO (Fig. 3), which considering the composition of the initial CO enrichment cultures, could benefit growth of both *Acetobacterium* species and hydrogenotrophic methanogens. H_2_ production by *Sporomusa* strains from CO was not shown before, but these bacteria have an important role in CO conversion in the mixed culture. A remarkable observation is that the *Sporomusa* species in CO‐P(12) can grow with 1.7 × 10^5^ Pa CO. Growth inhibition of *S. termitida* was observed for CO partial pressures higher than 0.4 × 10^5^ Pa (Breznak *et al*., 1988). Further research is needed to compare the enriched *Sporomusa* strain with other *Sporomusa‐*type strains.

## Experimental procedures

### Source of inoculum and medium composition

Granular sludge obtained from a 10‐L MOBB fed with syngas mixture (60% CO, 30% H_2_ and 10% CO_2_ (v/v)) (Pereira, 2014) was used as inoculum for batch incubations with CO. Cultures were prepared in 120‐ml serum bottles containing 30 ml of bicarbonate‐buffered mineral salt medium and sealed with butyl rubber stoppers and aluminium crimp caps. Medium was prepared as described by Stams *et al*. (1993). Carbon monoxide was used as the sole energy and carbon source and added to the bottles’ headspace to the desired final partial pressure using a syringe. All the assays were carried out at initial total pressure of 1.7 × 10^5^ Pa, for which a mixture of N_2_/CO_2_ (80:20 (v/v)) was added to the headspace when necessary. For assays with CO partial pressures higher than 1.3 × 10^5^ Pa, a phosphate‐buffered mineral salt medium was used and prepared as described by Alves *et al*. (2013b). Medium was reduced prior to inoculation with 0.8 mM (final concentration) sodium sulphide (Na_2_S·*x*H_2_O, *x* = 7–9). Cultures were monitored by measuring CO depletion and methane, hydrogen and acetate production.

### Batch assays

An overview of the experimental set‐up implemented in this study is illustrated in Fig. 4. To test the direct methanogenesis from CO, methanogenic sludge was incubated with the antibiotics vancomycin (v) and erythromycin (e) (bacterial growth inhibitors): CO‐v_50_e_100_ (50 μM vancomycin + 100 μM erythromycin) and CO‐v_100_e_100_ (100 μM of each antibiotic). Incubations without antibiotics were also performed. Bottles’ headspace contained 40% CO (pCO = 6.8 × 10^4^ Pa) as substrate, and cultures were incubated at 37°C. None of the incubations with antibiotics could use CO or produce methane. Incubations without antibiotics were successively transferred (10% (v/v)) to new bicarbonate‐buffered medium and fed with CO for 2 years with 40% CO, resulting in an enrichment series designated as **CO**(x) (where x stands for number of successive transfers). Since an early enrichment stage, the presence of sporulating cells in the CO cultures was evident (microscopic observation). An aliquot of culture CO(4) was pasteurized and used as inoculum to start the enrichment series **CO‐P(x)** (where P stands for pasteurization; x stands for number of successive transfers). Pasteurization procedure consisted in heating up the culture to 80°C for 20 min. Culture CO‐P(1) was transferred four times to fresh medium, and at this time, a second pasteurization was performed (culture CO‐P(4)). Pasteurization temperature was now increased to 95°C (for 20 min). In transfers immediately after each pasteurization, yeast extract and ethanol were used to stimulate growth, being removed in posterior transfers. CO was the sole carbon and energy source added (40%; pCO = 6.8 × 10^4^ Pa) in the next transfers (CO‐P(5) and CO‐P(6)). Then, the CO partial pressure in the headspace was raised gradually from 40% (pCO = 6.8 × 10^4^ Pa) to 100% CO (pCO = 1.7 × 10^5^ Pa) (CO‐P(7) to CO‐P(12)).

### Analytical methods

Gas samples were analysed by gas chromatography with a Bruker Scion 456‐GC (Billerica, MA, USA) with a thermal conductivity detector and equipped with two columns: a BR‐QPLOT column (30 m length, 0.53 mm internal diameter; film thickness, 20 μm) and a Molsieve packed column (13 × 80/100, 2 m length, 2.1 mm internal diameter). The Molsieve column was used to measure CO, H_2_ and CH_4,_ and argon was used as carrier gas at a flow rate of 30 ml min^−1^; temperatures in the injector, column and detector were 100, 35 and 130°C respectively. Volatile fatty acids (VFA), such as acetate, were determined by high‐performance liquid chromatography using an HPLC (Jasco, Tokyo, Japan) with a Phenomenex Rezex ROA – organic acid H+ (8%) column (300 × 7.8 mm). The mobile phase used was sulfuric acid (0.005 N) at a flow rate of 0.6 ml min^−1^. Column temperature was set at 60°C. Detection of VFA was made sequentially with an UV detector at 210 nm.

### DNA extraction and amplification

Five millilitres of well‐homogenized MOBB sludge, **CO**(x) and **CO‐P**(x) cultures was stored at −20°C. DNA extraction from these samples was performed using the FastDNA SPIN kit for soil (MP Biomedicals, Solon, OH) according to the manufacturer's protocol. The microbial 16S rRNA genes were amplified by PCR using a Taq DNA polymerase kit (Invitrogen, Carlsbad, CA). PCR programs and reactions mixtures used were as described elsewhere (Sousa *et al*., 2007), and all primers set were synthesized by Invitrogen. For 16S rRNA gene amplification for denaturing gradient gel electrophoresis (DGGE), primers set 1401r/968‐GCf was used for bacteria and 515‐GCr/A109(T)f for archaea (Sousa *et al*., 2007). The yield and size of PCR products were assessed by electrophoresis in 1% agarose gel (wt/vol), using a 1 kb extended DNA ladder (ThermoScientific, Waltham, MA, USA) and a green safe staining.

### DGGE analysis

DGGE analysis was performed using a DCode system (Bio‐Rad, Hercules, CA). For the purpose, gels of 8% (wt/vol) polyacrylamide (37.5:1 acrylamide/bis‐acrylamide) were used with a denaturing gradient of 30% to 50% for archaea and 30% to 60% for bacteria, with 100% of denaturant corresponding to 7 M urea and 40% (v/v) formamide. Electrophoresis ran in a 0.5 TAE buffer at 60°C for 16 h at 85 V. Posteriorly gels were stained with silver nitrate (Sanguinetti *et al*., 1994) and scanned in an Epson Perfection V750 PRO (Epson, Long Beach, CA, USA).

### 16S rRNA gene sequencing

Cloning and Sanger sequencing were performed using the methodologies previously described by Sousa *et al*. (2007). Similarity searches were performed using the NCBI BLAST search program within the GenBank database (http://www.ncbi.nlm.nih.gov/blast/) (Altschul *et al*., 1990). Illumina Miseq platform sequencing was performed at the Research and Testing Laboratory – RTLGenomics (Lubbock, TX). The MiSeq method used was the Illumina two‐step using universal primers for bacteria and archaea, 515f and 806r developed by Caporaso *et al*. (2011). After sequencing, the data were processed using the data analysis pipeline from RTL, which consists in two major steps, the denoizing and chimera detection step and the microbial diversity analysis step, as described in the company procedures.

### Nucleotide sequence accession numbers

The 16S rRNA gene sequences obtained in this study have been deposited in the European Nucleotide Archive (ENA) under the accession numbers LT671598–LT671603 (Sanger sequencing) and ERS1422865–ERS1422866 (Illumina MiSeq platform); these sequences can be viewed by following URL http://www.ebi.ac.uk/ena/data/view/PRJEB16760).

## Conflict of interest

None declared.

## Supporting information


**Fig. S1.** Bacterial and archaeal DGGE profiles of the enrichments **CO**(x) and **CO‐P**(x), where (x) corresponds to number of successive transfers (nomenclature in Fig. 4).
