# Supplementary material for: Enrichment of syngas‐converting communities from a multi‐orifice baffled bioreactor
Source: Microb Biotechnol. 2017 Nov 21;11(4):639–46. doi: 10.1111/1751-7915.12864 (PMC6011948; doi:10.1111/1751-7915.12864)
Supplement: Supplementary file 1 — Fig. S1. Bacterial and archaeal DGGE profiles of the enrichments CO(x) and CO‐P(x), where (x) corresponds to number of successive transfers (nomenclature in Fig. 4). [file MBT2-11-639-s001.doc]

**Enrichment of syngas-converting communities from a multi-orifice baffled bioreactor**

Ana L. Arantes^1,2^, Joana I. Alves^1^, Alfons J. M. Stams^1,2^, M. Madalena Alves^1^, Diana Z. Sousa^1,2^

***Supplementary material***


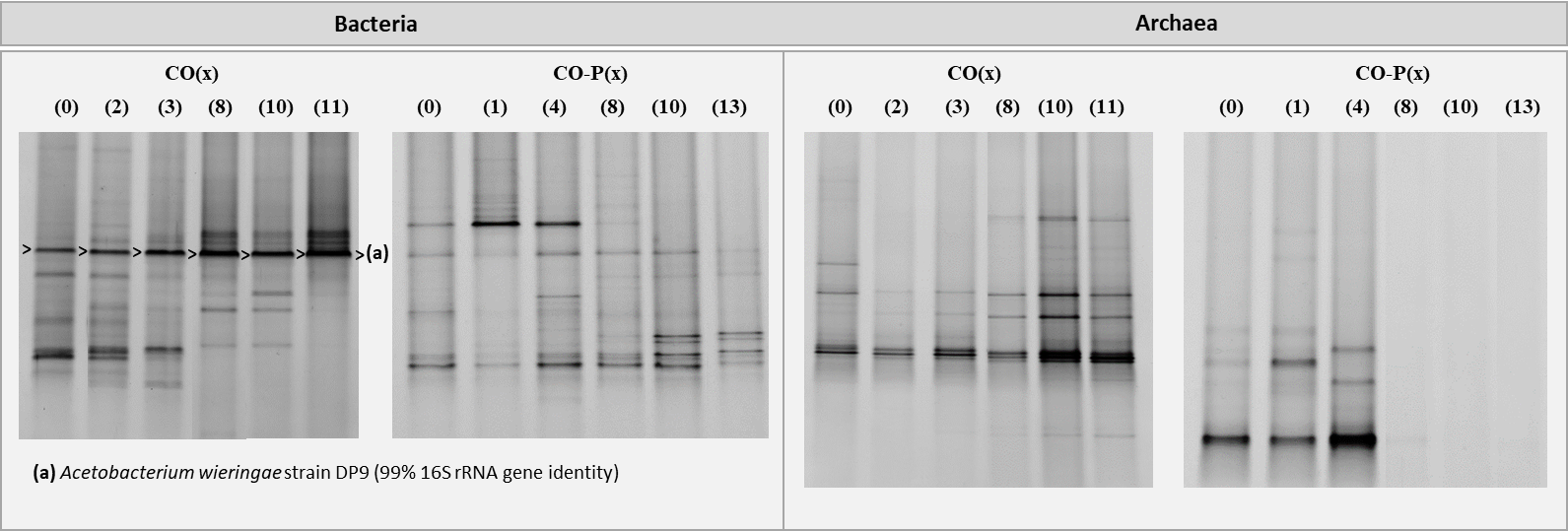


**Figure S1** – Bacterial and archaeal DGGE profiles of the enrichments **CO(x)** and **CO-P(x)**, where (x) corresponds to number of successive transfers (nomenclature in Figure 4).
